# Endemic Human Monkeypox, Democratic Republic of Congo, 2001–2004

**DOI:** 10.3201/eid1306.061540

**Published:** 2007-06

**Authors:** Anne W. Rimoin, Neville Kisalu, Benoit Kebela-Ilunga, Thibaut Mukaba, Linda L. Wright, Pierre Formenty, Nathan D. Wolfe, Robert Loshima Shongo, Florimond Tshioko, Emile Okitolonda, Jean-Jacques Muyembe, Robert W. Ryder, Hermann Meyer

**Affiliations:** * University of California, Los Angeles, California, USA; † National Institute of Biomedical Research, Kinshasa, Democratic Republic of Congo; ‡ Ministry of Health, Kinshasa, Democratic Republic of Congo; § National Institutes of Health, Bethesda, Maryland, USA; ¶ World Health Organization, Geneva, Switzerland; # Johns Hopkins Bloomberg School of Public Health, Baltimore, Maryland, USA; ** World Health Organization, Kinshasa, Democratic Republic of Congo; †† Kinshasa School of Public Health, Kinshasa, Democratic Republic of Congo; ‡‡ University of North Carolina, Chapel Hill, North Carolina, USA; §§ Bundeswehr Institute of Microbiology, Munich, Germany

**Keywords:** Human monkeypox, monkeypox virus, chickenpox, varicella-zoster virus, VZV, Democratic Republic of Congo, real-time PCR, hemagglutinin gene, dispatch

## Abstract

By analyzing vesicle fluids and crusted scabs from 136 persons with suspected monkeypox, we identified 51 cases of monkeypox by PCR, sequenced the hemagglutinin gene, and confirmed 94% of cases by virus culture. PCR demonstrated chickenpox in 61 patients. Coinfection with both viruses was found in 1 additional patient.

Monkeypox (MPX) virus is an orthopoxvirus that causes human MPX, a smallpoxlike disease reported in the African rainforests. Humans acquire the virus through direct contact with infected animals or patients (*1*). To determine whether MPX virus could potentially occupy the niche vacated by smallpox virus, the World Health Organization (WHO) conducted an active surveillance program during 1981–1986 in the Democratic Republic of Congo (DRC) and identified 338 MPX cases (67% confirmed by virus culture). Epidemiologic data led to the conclusion that MPX was a sporadic disease with a low potential for person-to-person transmission and that infection could not sustain itself in the human population (*1*). In the following years (1986–1995), only 13 MPX cases were reported (*2*). However, during 1995–1996, >500 cases of suspected MPX were reported, although only a small number were laboratory confirmed (*3*). In contrast to the earlier findings by WHO, the percentage of secondary cases was much higher (78%) and the mortality rate was much lower (1.5%). The question was raised as to whether a number of these cases were actually chickenpox, caused by the varicella-zoster virus (VZV), which is characterized by a high rate of secondary transmission and a low mortality rate (*4*). Although MPX is the only known severe orthopoxvirus infection today, the current dynamics of MPX infection are poorly understood and little information is available to improve WHO recommendations for the prevention of human MPX. We report laboratory data obtained from patients with suspected MPX infection.

## The Study

As part of the DRC Ministry of Health national disease surveillance program, 2,734 cases of suspected human MPX were reported from all 11 DRC provinces during January 2001–December 2004: 380 cases in 2001, 545 in 2002, 783 in 2003, and 1,026 in 2004. However, because civil war severely hampered surveillance activities, only 171 clinical specimens were obtained from 136 patients, who represent 4.9% of all reported cases. Ethical approval for this study was obtained from the Kinshasa School of Public Health, DRC, and the University of North Carolina, Chapel Hill, NC, USA.

All 171 specimens (crusted scabs and vesicle fluids) were inoculated onto MA104-cells by using standard procedures in a Biosafety Level 3 laboratory. Results yielded 56 MPX virus isolates from 48 patients (Table 1); scab and fluid specimens from the same patient were virus-positive for 8 patients. Identity of all isolates as MPX virus was confirmed by sequencing the entire open reading frame of the hemagglutinin (HA) gene.

**Table 1:** Orthopoxviruses and varicella-zoster virus, Democratic Republic of Congo, 2001–2004*

Specimen (no. patients)	MPX virus isolated	PCR result	
		Orthopoxvirus	VZV
Crusts (50)	16	18	21
Vesicle fluid (51)	19	21	24
Crusts and vesicle fluid (35)	8 and 5†	13‡	17‡
Total (136)	48	52	62

*MPX, monkeypox; VZV, varicella-zoster virus. †Virus was isolated from both specimens from 8 patients and from either specimen from 5 patients. ‡Both specimens were positive by PCR.

DNA of all specimens was analyzed by using the RealArt Orthopox LightCycler PCR kit (QIAGEN, Hilden, Germany), which amplifies sequences of the fusion protein gene present in all orthopoxviruses, including MPX virus (*5*). Subsequent melting-curve analysis enables further differentiation of either variola or nonvariola orthopoxviruses; however, specific identification of MPX virus is not possible. We amplified nonvariola orthopoxvirus sequences in 65 specimens from 52 patients (Table 1); all specimens from which MPX virus had been isolated were also positive by PCR. For all 13 patients from whom crusts and vesicle fluids were available, both specimens were PCR-positive.

Of 171 specimens examined by using RealArt VZV LC PCR (QIAGEN), 78 showed specific amplification that indicated that 62 patients had VZV infection (Table 1). Of 136 patients investigated, 1 was coinfected with both, MPX (PCR and virus isolation) and VZV (PCR). Coinfections were reported during the 1996–1997 outbreak (*3*) and in a patient who died in 2001 (*6*). Whether these coinfections resulted from simultaneous circulation of both viruses or to a mutual influence in the pathogenesis of either virus is not known.

Data on sex and age were available for 134 patients. The male-to-female ratio was approximately equal: 66 males and 68 females (Table 2). Ages ranged from 2 months to 54 years; average was 15.4 years (median 11.0). The average age of patients with confirmed MPX was 10.0 years (median 7.0 years), whereas the mean age of patients with VZV infection was 20.6 (median 17.0, p<0.001, t = 4.625, df = 109). That most MPX cases (94%) occurred in patients <25 years of age suggests that cross-protective immunity may still exist; a potential for continued immunity has been demonstrated (*7*). However, this difference could also reflect different exposures in adults >25 years of age.

**Table 2:** Age and sex distribution of patients with monkeypox or chickenpox, Democratic Republic of Congo, 2001–2004*

Age, y	No. cases investigated male/female, n = 134	MPX-positive male/female, n = 51	VZV-positive male/female, n = 61
<4	12/21	8/7	3/6
5–14	22/19	12/9	7/8
15–24	17/13	5/7†	10/5†
25–34	9/8	2/0	5/6
>35	6/7	1/0	5/6
Total	66/68	28/23	30/31

*MPX, monkeypox; VZV, varicella-zoster virus. †Coinfection with MPX and VZV in a 17-year-old female patient.

**Figure 1:**
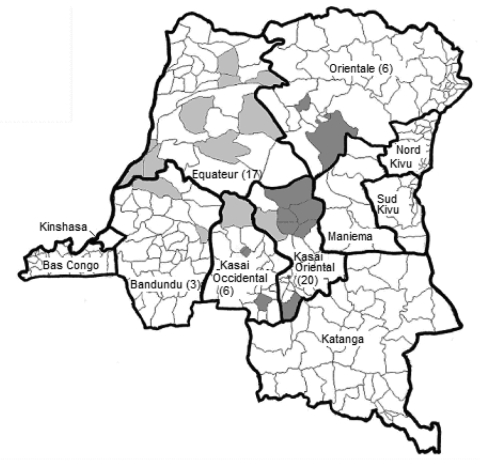
Distribution of 52 confirmed cases of human monkeypox (MPX) by health zone in the Democratic Republic of Congo (DRC), 2001–2004. The cumulative number of cases per province is in parentheses. Confirmed cases of MPX originated from a total of 26 different health zones located in 5 provinces of DRC; most (70.5%) were reported from Kasai Oriental and Equateur Provinces. Two groups can be differentiated on the basis of sequence of the hemagglutinin gene: light gray, group 1; dark gray, group 2. Note: The boundaries of the DRC health zones have since been redrawn. Although the health zones Tshopo and Mweka are not shown (located in provinces Orientale and Kasai Occidental, respectively), the general area is highlighted to represent MPX cases in these regions.

The DRC is divided into 512 administrative health zones that are within 11 provinces (Figure 1). Consistent with earlier reports (*1*), all MPX patients identified in this study lived mainly in small villages located in or near the tropical rainforest, where populations have ample opportunities for multiple close contacts with animals. No MPX cases have been identified in the area of Kinshasa. In some health zones, MPX virus and VZV circulate simultaneously (data not shown).

**Figure 2:**
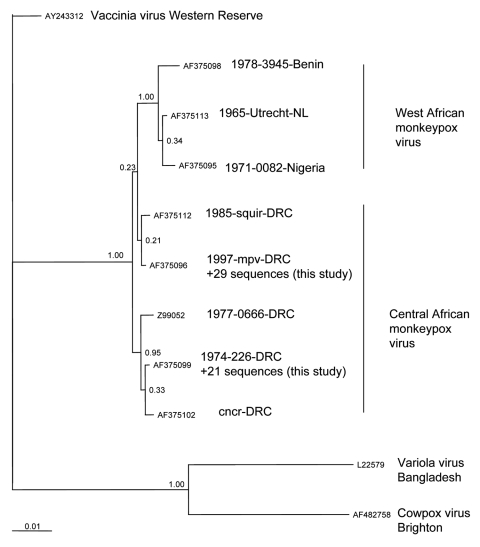
Phylogenetic inference relationships of the open reading frames encoding the hemagglutinin protein of selected strains of vaccinia, variola, cowpox, and monkeypox viruses and monkeypox virus isolates described in this study. Sequences used were cowpox virus Brighton (AF375089), variola virus Bangladesh (AF375129), vaccinia virus Lister (AY678276), monkeypox virus mpv 1997 (AF375096), mvp-squir (AF375112), mpv Zaire 77-0666 (Z99052), mpv-cncr (AF375102), mpv 74-226 (AF375099), mpv-082 (AF375095), mpv-utc (AF375113), and mpv-3945 (AF375098). ClustalW, version 1.83 (*10*), was used to generate amino acid multiple sequence alignments (pairwise gap opening = 35 and gap extension = 0.75; multiple alignment gap opening = 15 and gap extension = 0.30; Gonnet series). Each alignment was processed using RevTrans (*11*). Bayesian posterior probability inference of phylogeny used MrBayes, version 3.084. MrBayes settings for the best-fit model (GTR+I+G) were selected by hierarchies for the likelihood ratio test in MrModeltest 2.0 (*12*). Bayesian analysis was performed with MrBayes; the maximum likelihood model used 6 substitution types (nst = 6). Rate variation across sites was modeled by using a gamma distribution, with a proportion of sites being invariant (rates = invgamma). The Markov chain Monte Carlo search was run for 1 million generations; trees were sampled every 100 generations (the first 4,000 trees were discarded as burn-in). NL, the Netherlands; DRC, Democratic Republic of Congo.

The entire sequence (942 nt) of the open reading frame of the HA gene was determined (*8*) for 48 virus isolates derived from 48 patients after PCR amplification. In addition, the HA gene sequence was successfully determined directly from lesion material of 2 MPX patients from whom no virus isolate was available; for a third patient, only a part of the HA sequence could be sequenced. All HA nucleotide sequences were deposited in GenBank (accession nos. DQ443476 through DQ443525). Sequencing identified 2 distinct groups consisting of 29 and 21 identical sequences, which differed by a single nucleotide. Geographic data analysis demonstrated that this difference was distributed consistently between patients living in east and west DRC (Figure 1). There were no sequence differences in the MPX virus hemagglutinin gene (942 nt) of isolates from the same patient. The 2 sequences determined here were identical to sequences reported previously (*9*) from MPX patients in the DRC (AF375099 and AF375096, respectively). The sequences represent 2 of 5 branches of the Central African MPX virus clade (Figure 2), which is distinguishable from a second clade comprising isolates from 1) West Africa, 2) outbreaks in primate-holding facilities in Europe and the United States, and 3) the recent 2003 US outbreak.

For our study, we used 2 commercially available kits to amplify orthopoxvirus and VZV sequences. The assays contain an internal control to monitor inhibition and use 4 positive controls to enable quantification of the amount of input genomic viral DNA. On the basis of our results, one can calculate that MPX lesion material usually contains several million viral genomic copies (data not shown). Whereas WHO surveillance confirmed 67% by virus culture (*1*), we isolated MPX virus in 94% of the MPX patients who had positive PCR results. The rather high rate of virus culture–confirmed cases points to the robust and highly concentrated amounts of virus in lesions; virus remained viable in lesions over an extended period despite suboptimal collection and transportation conditions.

## Conclusions

Among 136 patients, 51 (37.5%) had laboratory-confirmed MPX infection, 61 (44.8%) had laboratory-confirmed VZV infection, and 1 (0.7%) had coinfection. MPX virus is considered to be the most important orthopoxvirus infection in humans because it causes disease clinically indistinguishable from smallpox. Recent outbreaks of MPX in the United States (*13*), the Republic of Congo (*14*) and Sudan (*15*) highlight the capacity of this virus to appear where it has never before been reported. Because clinically, MPX is often confused with chickenpox, laboratory diagnosis is critical to determine whether a suspected case is indeed MPX. In our study, we identified the causative agent for a rash-causing illness in 83% of all patients. We have recently strengthened the MPX surveillance program to improve understanding of the epidemiology of human MPX.
